# Viral Biomarkers in Chronic HBeAg Negative HBV Infection

**DOI:** 10.3390/genes9100469

**Published:** 2018-09-27

**Authors:** Emilia Hadziyannis, Andreas Laras

**Affiliations:** Second Department of Medicine and Laboratory, Hippokrateio Hospital, National and Kapodistrian University of Athens, Athens 11527, Greece; alaras@med.uoa.gr

**Keywords:** HBV, biomarker, HBV DNA, HBV RNA, HBsAg, HBcrAg, anti-HBe

## Abstract

Viral biomarkers are important tools for monitoring chronic hepatitis B virus (HBV) hepatitis B early antigen (HBeAg) negative infection, both in its natural course as well as during and after treatment. The biomarkers consist of antibodies against viral epitopes, viral proteins, and molecular surrogate markers of the quantity and transcriptional activity of the stable episomal HBV covalently closed circular DNA (cccDNA) which is located in the nuclei of the infected hepatocytes. HBV deoxyribonucleic acid (DNA) or else viral load measurement in plasma or serum is a marker of HBV replication of major clinical importance. HBV DNA is used for staging and treatment monitoring as described in international scientific guidelines. Quantification of HBV antigens, mainly hepatitis B surface antigen (HBsAg) as well as Hepatitis B core related antigen (HBcrAg), play an important yet secondary role, especially in cases of low or undetectable HBV DNA and has been evaluated for the classification of the inactive carrier state, as a predictor of subsequent HBsAg clearance, treatment outcome, and development of hepatocellular carcinoma (HCC). The measurement of the replicative intermediate HBV RNA in serum is currently evaluated and may also prove to be a significant biomarker particularly in patients treated with nucleot(s)ide analogs. This review focuses on the viral biomarkers mentioned above and their role in HBV, HBeAg negative, infection.

## 1. Introduction

Chronic liver inflammation, or chronic hepatitis, is a common disease and a worldwide public health issue. Although, nonalcoholic fatty liver disease (NAFLD) is considered the most rapidly and continuously increasing cause of chronic hepatitis, chronic viral hepatitis is still of major importance, concerning approximately 330 million people worldwide. Specifically, chronic hepatitis B (CHB) affects about 240 million people and although the diagnostic tools are widely available, is still under-diagnosed [1]. 

**Figure 1:**
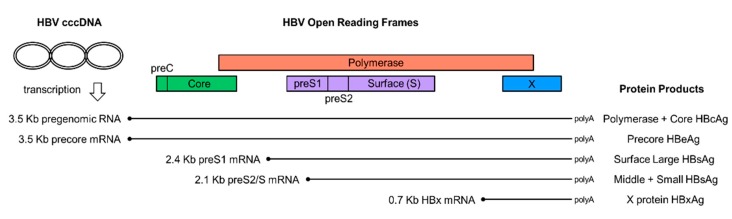
Hepatitis B virus (HBV) gene expression. The HBV covalently closed circular DNA (cccDNA) serves as the template for the transcription of the pregenomic RNA and subgenomic messenger RNA (mRNA) transcripts (shown as thin lines), aligned to a linear depiction of the viral open reading frames (shown as open boxes). The corresponding protein products for each of the major HBV transcript are listed on the right. HBcAg: HBV core antigen; HBeAg: HBV early antigen; HBsAg: HBV surface antigen; HBxAg: HBV X protein.

The diagnosis and follow up of chronic hepatitis B virus (HBV) infection relies on laboratory viral biomarkers. There are two major categories of HBV biomarkers. The first one is serology, a term comprising the detection and quantification of viral antigens and viral specific antibodies and the second is nucleic acid testing (NAT) for the detection and quantification of HBV genome and its RNA transcripts [2] (Figure 1).

Both serology and NAT are in routine use, not only for the diagnosis of chronic and acute HBV infection, but also for monitoring chronic HBV infection with or without treatment. By definition, HBV serology for hepatitis B early antigen (HBeAg) and the corresponding antibody, is imperative for the specific diagnosis of HBeAg negative CHB. Moreover, serological and molecular biomarkers are used for the identification of the HBeAg negative inactive carrier (IC) state, for treatment initiation and monitoring, and as predictors of HBV and liver related events [3,4]. 

## 2. Viral Biomarkers

### 2.1. Serology

#### 2.1.1. Antigen and Antibody Detection

The first identified biomarker of HBV infection was the hepatitis B surface antigen (HBsAg), formerly named Australia antigen. Its discovery by Blumberg was the most important milestone that led to the diagnosis, prevention, and treatment of hepatitis B [5]. The detection of HBsAg remains the principal diagnostic tool of HBV infection. HBsAg is produced in excess in HBV infected hepatocytes and circulates in high quantities in the serum [6], consequently, it is a very sensitive as well as specific biomarker for HBV infection. The patient’s profile, based on the results of the detection of HBsAg combined with the measurement of the respective anti-HBs antibodies plus the detection of anti-HBc antibodies (total and IgM), is adequate for the diagnosis of acute and for the screening of chronic HBV infection. Once the diagnosis of chronic HBV infection is made, testing for HBeAg and the corresponding anti-HBe antibody is mandatory and is usually performed by the use of the same methodology, as the previously mentioned serological markers.

Nowadays, extremely sensitive immunoassays on automated analyzers are used for HBV serology based on chemiluminescence (CLIA) and electrochemiluminescence technology (ECLIA) [7,8]. In some cases, however, the increased sensitivity of the HBsAg assays may lead to false positive results. Thus, in patients with HBsAg index values close to the cut off and with inconsistent other serological markers, verification of HBsAg positivity with a confirmatory assay is recommended [9] HBV antibodies and HBeAg detection are also performed on these automated immunoassay systems [10,11,12].

In many laboratories, standard enzyme-linked immunoassay (ELISA) is still used for HBV serology as it is an inexpensive alternative that does not require instrumentation other than a microplate reader (photometer). Commercial ELISAs demonstrate high sensitivity (>99%) and acceptable specificity (>95%) for HBsAg [13], but positive results with low absorbance need to be confirmed. Also, in the case of rheumatoid factor positivity there may be positive or negative interference in these assays [14]. The concordance between ECLIA and ELISA has been found to be 97.05% for HBsAg, 92.62% for anti-HBs, 100% for HBeAg, 76.75% for anti-HBe, and 58.67% for anti-HBc. Particularly in patients with HBeAg and anti-HBe coexistence, the concordance for HBeAg detection was found to be 45.83% and for anti-HBe 79.17%. The discrepancies of the assays were attributed mainly to differences in their sensitivity [15]. 

Screening for HBV is also feasible with rapid tests at the point of care (POC). Point of care serology is available as a single test for HBsAg only or in the form of multiple serological markers detection e.g., for hepatitis B, hepatitis C, human immunodeficiency virus (HIV), and syphilis [16]. Rapid tests for HBsAg use capillary blood specimen obtained from a finger stick, are easy to execute and have a sensitivity of greater than 90% and specificity of over 99.5%. On the other hand, the performance of anti-HBs rapid tests is not optimal, with the major limitation being their low sensitivity [17]. Anti-HBc POC tests are not available.

#### 2.1.2. Hepatitis B Surface Antigen Quantification 

HBsAg is translated from messenger RNAs (mRNAs) transcribed from covalently closed circular DNA (cccDNA) and/or from HBV sequences integrated in the host genome. The quantification of HBsAg in the past was performed for research purposes by serial serum dilutions that allowed the determination of its titer with qualitative assays [18]. Currently, HBsAg quantification (qHBsAg) in serum is measured in international units per milliliter (IU/mL) on the same instruments that are used for routine serology, but with different reagents and in most cases with on board dilution of the sample [19,20,21]. The lower limit of HBsAg detection for the majority of the quantitative assays is 0.05 IU/mL in undiluted samples, which is higher than the cut off of the respective qualitative assays.

Automated HBsAg quantification has been proven to be highly reproducible and the results between different analyzers show very good correlation [19]. Nevertheless, it is recommended to employ the same assay for monitoring, since differences, especially in the low ranges, might be of importance. The concentration of HBsAg in serum has been studied in every aspect of HBV infection and the test is commercially available and marked for in vitro diagnostics (IVD).

#### 2.1.3. Hepatitis B Early Antigen Quantification 

HBeAg quantification (qHBeAg) is not yet available as a standardized commercial assay. Nevertheless, it has been evaluated, by in house methodologies, as a possible biomarker in HBeAg positive infection [22]. Monitoring of qHBeAg has been found to be helpful for the prediction of response to treatment and sustained HBeAg seroconversion [23,24,25,26]. There are ongoing efforts for the standardization of qHBeAg assays, which is imperative for the generation of robust results and potential clinical use of the test [22,27]. Evidently, in established HBeAg negative hepatitis, qHBeAg is not applicable.

#### 2.1.4. Hepatitis B Core Related Antigen Quantification 

Hepatitis B core related antigen (HBcrAg) is a rather new marker, that incorporates the quantification HBcAg, HBeAg, and core related protein p22 (p22cr) in one test. These three proteins are all products of the precore/core HBV gene and share the same 149 amino acid sequence. Obviously, patients positive for HBeAg, which is included in the assay, are found with higher HBcrAg levels than their HBeAg negative counterparts in whom HBcrAg is undetectable in many cases. This test has been used for research only and was first available only in one automated analyzer (Lumipulse, Fujirebio), with lower limit of detection (LOD) being high at 2 logs U/mL and lower limit of quantitation (LOQ) at 3 log U/mL [28,29]. The quantification of the denatured HBcr proteins is accomplished in reference to a standard curve of known quantities of HBeAg recombinant peptides (1 U/mL = 10 fg/mL recombinant HBeAg) [30,31].

**Figure 2:**
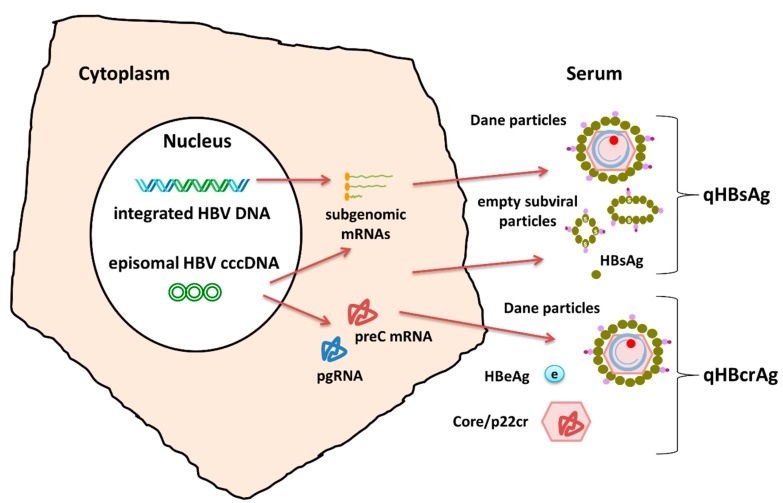
Serum HBsAg reflects the transcriptional activity of both cccDNA and integrated HBV sequences. preC mRNA: precore messenger RNA; pgRNA: pregenomic RNA; qHBcrAg: quantitative HBV core related antigen.

There is ongoing and expanding research concerning its use as a potential biomarker in both HBeAg positive and negative phase of the infection, since HBcrAg reflects the transcriptional activity of intrahepatic cccDNA more accurately than qHBsAg, which is produced not only by cccDNA but also from integrated HBV DNA sequences (Figure 2). HBcrAg levels have been associated with intrahepatic cccDNA levels, even in serum HBV DNA negative patients [32].

On the whole, HBcrAg levels display a strong correlation with serum HBV DNA and statistically significant but moderate (r = 0.78) correlation with qHBsAg, which is even less pronounced in the HBeAg negative state [30].

### 2.2. Molecular Markers (Nucleic Acid Testing)

#### 2.2.1. Hepatitis B Virus Deoxyribonucleic Acid

Hepatitis B Virus DNA, the genomic nucleic acid of the virus and internal constituent of the core particle, was identified by 1975 and has been sequenced since 1980 but was not used as a biomarker until the molecular techniques advanced to a level permitting their use in specialized clinical laboratories. Actually, until then, the presence of HBeAg was solely associated with active viral replication and active liver disease. With the application of low sensitivity molecular hybridization techniques for serum HBV DNA measurement in HBeAg negative patients, it became clear that although the viral load is higher in HBeAg positive individuals, there is a proportion of HBeAg negative patients who have detectable HBV DNA and active hepatitis [33,34,35]. 

Subsequently, with the advent of polymerase chain reaction (PCR) assay for nucleic acid amplification and branched DNA for hybridized signal amplification, HBV DNA measurements became an important viral biomarker. Viral load measurement is currently accomplished with concurrent detection and quantification of HBV DNA in plasma or serum by sensitive real time PCR assays. Several manufacturers provide real time PCR platforms and reagents approved for patient diagnostic use (IVD marked). Some examples of the most commonly used assays are the Roche COBAS TaqMan HBV Test, Abbott RealTime HBV Assay, Siemens VERSANT HBV DNA, and Qiagen artus HBV RG PCR. These and even newer assays demonstrate good reproducibility with low imprecision, as measured by the coefficient of variation (CV 0.5–4%) and excellent correlation between the different systems (r > 0.9) [36,37]. Moreover, point of care NAT instrumentation is expanding their menu options to include HBV DNA testing. The current available HBV DNA POC assays are used for research only. Their performance appears to be similar to the assays used in central laboratories on automated or semi-automated analyzers, thus it is probable that some POC NAT HBV DNA tests and instruments are going to be approved for IVD use in the near future [38].

According to the American Association for the Study of Liver Diseases (AASLD) guidelines for treatment of chronic hepatitis B and the European Association for the Study of the Liver (EASL) clinical practice guidelines, the required sensitivity for HBV DNA detection is 10 IU/mL [39,40]. The PCR assays that are commonly used demonstrate aLOD which is usually lower than 10 IU/mL and a higher limit of quantification (LOQ). Therefore, a not quantifiable but detectable HBV DNA result is probable and both laboratory and clinical personnel need to be aware of this possibility. The genetic variability of different HBV genotypes and subgenotypes appears to have minimal, if any, effect in HBV DNA quantification. Nonetheless, mutations in the primer or probe binding regions may lead to underestimation of the viral load [41].

HBV viremia can be measured not only in fresh blood, but also in dried blood spots (DBS) of whole blood collected by venipuncture or capillary blood collected by finger puncture. The DBS specimens are sent to a central laboratory where they are extracted and tested with conventional HBV DNA techniques. This approach, although not IVD marked as yet, could be important in resource restricted areas with limited access to NAT. In a recent meta-analysis of 12 studies meeting the inclusion criteria, the pooled estimate of sensitivity was 95% (95% confidence interval (CI): 83–99) with a higher specificity at 99% (95% CI: 53–100) for HBV DNA detection in DBS. The correlation with serum assays was reported in five studies and was found to be strong (r = 0.6–0.96) but the LOD was higher for DBS (914–2000 IU/mL). DBS sample storage conditions do not seem to affect the results of HBV DNA detection [42].

The measurement of HBV DNA in oral fluid samples, a different and slightly cheaper approach for the detection of active viral replication, has been used and evaluated in few studies [43,44,45,46]. This approach lacks in sensitivity particularly in the HBeAg negative infection and does not appear to be an appropriate alternative.

Additionally, recent advances in technology, made possible the direct detection and quantification of HBV cccDNA in serum, which is released into circulation due to the destruction of infected hepatocytes [47]. There is still need of detailed and extensive studies and of course wider availability of the latest technology, in order to start considering cccDNA in serum as a robust clinical biomarker.

#### 2.2.2. Hepatitis B Virus Genotype

HBV is classified in at least 10 genotypes (A to J) based on genetic divergence of more than 8% and further in subgenotypes within HBV genotypes with divergence of >4%. HBV genotypes and subgenotypes have distinct geographical distribution. There is increasing evidence of association of genotypes with disease progression and the pathogenesis of HBV infection [48].

The gold standard method for the identification of HBV genotype is whole genome sequencing followed by phylogenetic analysis [49], but most commonly, sequence of the preS-S/pol gene with comparison (sequence alignment) to HBV genotype consensus sequences or the line probe assay, are used [50,51]. It is of interest that mutations in the precore region of HBV, confirmed by sequencing, prevent the production of HBeAg (mainly the G1896A substitution), and are prevalent in HBeAg(-) patients with active liver disease and HBV genotypes D, C, and B [52,53].

The same methods that are applied for genotyping, are also used for the detection of genetic resistance of HBV to NAs [54].

Although different HBV genotypes seem to have diverse biological behavior, HBV genotyping is not used as a routine viral biomarker most probably due to their already recognized geographical distribution. 

#### 2.2.3. Hepatitis B Virus Ribonucleic Acid

The HBV cccDNA nuclear episome, is the template for transcription by host enzymes producing the viral mRNA transcripts which are translated into viral proteins, as well as the 3.5 kb pregenomic (pg) RNA which is the template for reverse transcription. Synthesis of the minus (–) strand DNA and synchronous degradation of the RNA pregenome is followed by partial synthesis of variable length plus (+) strand leading to the production of mature viral particles containing the relaxed circular (RC) DNA genome. Virions are either enveloped and secreted or recycled to the nucleus to increase the available cccDNA pool. However, HBV RNA can be detected in the serum of either HBV DNA positive or negative patients in the natural course of CHB and under treatment [55,56,57,58,59,60,61,62]. Several investigators have confirmed that at least the majority of serum HBV RNA is pgRNA encapsidated in virus-like particles [62,63,64], however other HBV RNA species have been detected both in vivo and in vitro [64,65,66,67].

Serum HBV RNA is currently under evaluation as a surrogate non-invasive marker, for monitoring intrahepatic cccDNA transcriptional activity. Detectable HBV RNA in the serum is indirect evidence for cccDNA persistence. A number of different experimental methods for the quantification of intrahepatic and serum HBV RNA are utilized based on quantitative real-time RT PCR assays, however there is no consensus on a single technique and commercial available assay for the detection of HBV RNA. Units used in this experimental setting are either HBV RNA copies/mL or IU/mL of serum, independently standardized by investigators.

## 3. Clinical Significance of Viral Biomarkers in HBeAg(-) Infection

### 3.1. Biomarkers for the Diagnosis of Chronic HBeAg Negative Hepatitis B Virus Infection

The diagnosis of chronic hepatitis B virus (HBV) infection is made based on the presence of hepatitis B surface antigen (HBsAg) in serum over six months or a specific serological pattern that includes positive HBsAg, negative respective antibody (anti-HBs) and positive total core antibody (anti-HBc) with negative IgM anti-HBc (Table 1).

The differentiation between HBeAg positive and negative chronic infection is made with the use of two additional serological markers namely the HBeAg and the respective antibody. Depending on how close to HBeAg seroconversion the patient is, HBeAg negative patients may be anti-HBe negative or positive. If a patient was known to be HBeAg positive and recently seroconverted, serial determinations are necessary to establish that the patient transcended to the HBeAg negative stage of chronic HBV infection.

Occasionally, a non-diagnostic serological profile with negative HBsAg and anti-HBs but positive anti-HBc is encountered. Although in the majority of these cases this profile is due to past HBV infection there is a possibility of occult chronic HBV infection. Occult HBV infection is defined as HBsAg negative and HBV DNA positive in serum and/or liver [68,69,70,71,72,73]. In circumstances where patients positive for anti-HBc only, are going to receive immunosuppressive therapy it is imperative to be tested for HBV viremia with NAT (HBV DNA). Immunosuppression may lead to HBV reactivation, thus prophylactic antiviral treatment is implemented [74,75]. 

### 3.2. Biomarkers for the Identification of the Inactive Carrier State

HBeAg negative patients with chronic HBV infection are categorized in two main states. In the inactive carrier state (recently renamed by EASL as ‘HBeAg negative chronic infection’), there is limited viral replication and liver inflammation [40]. The inactive HBV carrier presents with normal alanine aminotrasferase (ALT) levels (less than 35 U/L in males and less than 25 U/L in females) and low (<2000 IU/mL) or undetectable HBV DNA. Although the above cut-offs are based on international guidelines, the characterization of a patient as inactive carrier requires serial determinations of ALT and HBV DNA, every 3–4 months for the first year and subsequently every 6 months. This approach is imperative due to the fluctuations of HBV DNA and ALT that occur in patients at the second state of HBeAg negative infection with active hepatitis [39,40,76]. 

Since close follow up and serial testing of two biomarkers is essential for the identification of inactive carriers, the addition of a third biomarker has been evaluated. Given that the majority of inactive HBV carriers, have lower HBsAg levels in serum than patients with active inflammation [77,78], the first biomarker that was studied was qHBsAg. In a thorough study on HBV genotype D infection, qHBsAg values at a single time point of less than 1000 IU/mL in combination with HBV DNA < 2000 IU/mL and normal ALT demonstrated a diagnostic accuracy (DA) of 94.5% for the identification of the inactive carrier state, compared to monthly monitoring with the latter two biomarkers for one year. More specifically, the sensitivity of a single three-markers measurement was 91.1%, a specificity of 95.4%, a positive predictive value (PPV) of 87.9%, and a negative predictive value (NPV) of 96.7% [79]. Similar results have been observed in other HBV genotypes. In one study of 1068 Taiwanese HBeAg negative patients that had been diagnosed as HBV carriers, infected with HBV genotype B or C, the relationship between HBsAg level > 1000 IU/mL and the development of HBeAg-negative hepatitis in 13 years follow up was found to be significant (hazard ratio (HR) = 1.5, 95% CI = 1.2–1.9) and HBsAg < 1000 IU/mL in combination with low HBV DNA and ALT were found to be useful for identifying minimal-risk HBV carriers [80]. In the REVEAL cohort, in patients infected with B or C genotype, the combined testing with the same HBsAg cut-off, showed diagnostic accuracy for IC of 78% [81]. This HBsAg threshold of 1000 IU/mL seems to be the most reliable one for the differential diagnosis of CHB and the inactive carrier state [82]. The use of a lower qHBsAg cut-off e.g., 100 IU/mL for increased specificity, results in significant decrease in sensitivity (35%) [83]. Besides qHBsAg, the addition of liver stiffness measurement (LSM) as a fourth parameter, with a cut off of 6.2 kPa further improves the diagnostic accuracy of testing ALT, HBV DNA, and qHBsAg in a single time point, showing 100% specificity, 96% sensitivity, 100% PPV, 92% NPV, and 97% DA for the identification of ICs [84]. 

Recently, it was suggested that serum HBcrAg is more accurate than qHBsAg for the identification of inactive carriers, regardless of hepatitis B virus genotype [85]. In this study, the diagnostic accuracy of HBcrAg ≤ 3 logU/mL combined with HBV DNA ≤ 2000 IU/mL was 87% for genotype D, lower for genotypes F or H (73%) but higher for genotypes A and E (91 and 94%).

Some patients who display HBeAg negative serological profile with normal ALT, low viremia and/or qHBsAg higher than 1000 IU/mL, have a benign course of the infection and a proportion of them fulfill the criteria of IC in later time. In a recent study with such population included, the combined qHBsAg and HBV-DNA quantification had a diagnostic accuracy of 65.4% and 100% NPV for the identification of ICs. In this study HBV-DNA ≤ 2000 IU/mL and HBcrAg < 3 log or HBV DNA ≤ 2000 IU/mL and total anti-HBc ≤ 16,937 IU/mL had higher and almost the same DA around 86.5% and their diagnostic performance was improved by combing HBV-DNA ≤ 2000 IU/mL, HBcrAg ≤ 3 log and total anti-HBc ≤ 16,937 IU/mL (DA 89.5%, sensitivity 93%, specificity 84.8%, PPV 88.9%, NPV 90.3%) [86]. Total anti-HBc was quantified by a double-antigen sandwich immune-assay, calibrated using WHO standards.

The biological importance and clinical relevance of serum HBV RNA during the natural course of HBV infection and the differentiation between HBeAg negative carriers and active infection remain unclear. An early study suggested that HBV RNA in serum could be a useful marker for the recognition of the stages of chronic HBV infection [61]. Recently, HBV RNA levels were found to be independently associated with HBeAg status, serum ALT, HBV genotype, and the presence of BCP variants, in a large multiethnic cohort including 122 HBeAg(-) individuals (80% HBV genotype D) [87]. In another study including 24 HBeAg(-) patients (genotype B or C), it was shown that serum HBV RNA levels best correlated with intrahepatic HBV RNA levels, reflecting cccDNA transcriptional activity, rather than with intrahepatic cccDNA itself. The correlation with cccDNA, observed in HBeAg(+) patients, was not found in the HBeAg(-) group. No correlations between liver injury or histopathology and HBV-RNA levels were observed, nonetheless, the ratio of HBsAg to serum HBV-RNA was found to be the highest in IC patients [88]. Similarly, in a study of CHB patients, the correlation between serum HBV RNA and intrahepatic cccDNA levels was reported to be dependent on the serostatus of HBeAg and was not found in HBeAg(-) patients [89]. Furthermore, in an investigation that included HBeAg(-) negative subjects, the authors proposed the arithmetic addition of serum RNA on serum DNA levels in order to most accurately reflect intrahepatic cccDNA levels. However, this heterologous combination also failed to produce a correlation between serum RNA and liver cccDNA in the group HBeAg(-) chronically infected individuals [90]. Thus, concerning HBeAg(-) CHB, it is not clear whether or not and in what context serum HBV RNA can serve as a biomarker during the natural course of infection. Although correlation with intrahepatic cccDNA levels is poor, serum HBV RNA reflects viral activity at some level, specifically the transcriptional activity of cccDNA. However, its biological and clinical significance in the progression and pathogenesis of HBV infection require further investigation.

### 3.3. Biomarkers for the Prediction of Spontaneous HBsAg Clearance

Since 0.4–2.3% of HBe Ag negative inactive carriers clear HBsAg yearly [91,92,93], AASLD in 2018 suggested as practice guidance that HBeAg negative ICs should be tested annually for HBsAg [76].

In mostly Asian studies of patients infected with HBV genotypes B and C, it has been shown that the levels and kinetics of HBsAg in serum are predictors of subsequent spontaneous HBsAg loss. In one of the first studies, a threshold of baseline qHBsAg ≤ 100 IU/mL had 75% sensitivity and 91% specificity to predict subsequent HBsAg seroclearance, which was not associated with baseline serum HBV DNA [94]. Very low qHBsAg < 10 IU/mL was found to be an excellent predictor of its clearance, with a hazard ratio (HR) 13.2 compared to levels above 1000 IU/mL [80]. In another study, a baseline qHBsAg < 200 IU/mL resulted in a sensitivity of 84.2% and specificity 73.4% and qHBsAg kinetics, that is an annual 0.5 log reduction, in a sensitivity of 62.8% and 88.7% specificity for the prediction of HBsAg clearance, in a three year follow-up period [95]. More recently, in a cohort of patients with 3.08% annual HBsAg clearance rate, baseline qHBsAg levels predicted HBsAg loss (AUROC 0.965 (95% CI, 0.947–0.980)), with baseline levels < 10 IU/mL showing diagnostic an accuracy of 93.4%, a sensitivity of 87.2%, a specificity of 94.8%, a positive predictive value of 79.1%, and a negative predictive value of 97.0% [96]. In addition, a scoring system for the prediction of HBsAg seroclearance in HBeAg-seronegative chronic hepatitis B patients with genotype B or C infection has been proposed incorporating baseline qHBsAg and HBV DNA levels [97].

In a recent study, in patients infected mainly with HBV genotype D and 4.6% annual serum HBsAg clearance, the combination of qHBsAg ≤ 100-IU/mL with HBV-DNA ≤ 200-IU/mL exhibited good performance (87.5% DA, 84.2% sensitivity, 88.2% specificity, 66.7% PPV, and 95.2% NPV) for the prediction of HBsAg loss. Baseline q HBsAg levels were independently correlated with HBsAg clearance and were significantly lower (median 0.75 vs. 2.81 log10 IU/mL, *p* < 0.001) in patients who cleared HBsAg. Yearly decline of qHBsAg was also found to be a predictive factor being higher in patients who cleared HBsAg (median 0.22 vs. 0.020 log10 IU/mL/year, *p* < 0.001). HBcrAg was not found to be a predictor of HBsAg loss [86]. 

### 3.4. Treatment Monitoring

The clinical target of treatment in HBeAg negative hepatitis is to stop the progress of liver disease to cirrhosis and/or HCC. According to current guidance, therapy in HBeAg negative hepatitis is recommended in patients with elevated ALT ≥ 2 × upper limit of normal (ULN) and HBV DNA ≥ 2000 IU/mL and in cirrhosis irrespective of ALT [76]. These patients are in an immune active state with liver inflammation, viral replication, and transcriptional activity or they already have severe liver damage. The preferred regimen is an antiviral nucleos(t)ide analog (NA) with high genetic barrier (entecavir-ETV, tenofovir-TDF, and tenofovir alafenamide-TAF). Pegylated interferon (Peg IFN), which is not safe in cirrhotics and contraindicated in patients with decompensated cirrhosis, is used sometimes in patients with mild to moderate disease. Older NAs, in which the development of resistance is a major issue, are still used in some cases or in some countries due to their lower price.

The measurable goals of treatment are biochemical response with normalization of ALT levels in serum with viral remission with undetectable HBV DNA (complete viral response) in blood and ultimately functional cure which corresponds to HBsAg clearance. HBV DNA is the crucial biomarker used for monitoring the virologic response in treatment with NAs (Table 2). 

HBV cccDNA is responsible for viral persistence during prolonged antiviral therapy [98,99] and the production of pg HBV RNA reflects cccDNA transcriptional and replicative activity. Long term treatment with NAs results in suppression and undetectability of serum HBV DNA levels, by the inhibition of reverse transcription. This process does not directly affect the transcriptional activity of cccDNA and the production of pgRNA, mRNAs, and viral proteins is continued by the residual intrahepatic cccDNA [100]. Thus, HBV RNA pregenomes may accumulate in the hepatocytes during treatment and be packaged and exported in virion-like particles in the serum. It was firstly shown that, in patients receiving lamivudine therapy, HBV RNA becomes detectable in serum during treatment and that is inhibited by interferon-alpha (IFN-α) [59,101]. It has also been postulated that serum HBV RNA might be a predictor of early emergence of viral resistance mutations during NA therapy [55,102]. In one fairly recent study that included 12 HBeAg(-) NA treated patients, a positive correlation between the levels of serum HBV RNA and HBV DNA as well as with qHBsAg was demonstrated and the HBV RNA kinetics of HBeAg(-) and HBeAg(+) patients who achieved seroconversion were found to be similar [65]. More recently, in a study on entecavir treated patients, including 22 HBeAg(-) with genotype B or C HBV infection, it was shown that serum HBV RNA levels reflect intrahepatic viral activity and are associated with liver histopathology [103]. It is thus obvious that more studies are needed in HBeAg(-) infection in order to establish a possible role for HBV RNA in serum for treatment monitoring.

#### 3.4.1. Predictive Biomarkers for the Response to Pegylated-Interferon

Although only a limited number of HBeAg-negative patients receive pegylated-IFN (peg-IFN), several parameters have been identified as baseline predictors of response to this treatment. Younger age, female sex, higher ALT levels, and lower HBV DNA levels have been associated with greater probability of a sustained virological and biochemical response [104]. HBV genotypes appear to have an impact on sustained response (SR), with HBV genotype D infected patients being less likely to attain biochemical and virological remission enduring one year after treatment, compared to genotype B (odds ratio (OD): 3.69, *p* = 0.003) or genotype C (OD: 5.46, *p* < 0.001). It is also known that treatment with peg-IFN induces a decrease in qHBsAg which is much greater than in NA treatment [105]. Furthermore, baseline pretreatment qHBsAg titer < 1250 IU/mL has also been associated with sustained off-treatment viral response [106]. 

On treatment monitoring of HBV DNA and HBsAg have been proven to be essential since the negative predictive value for SVR is 100% when no decrease in qHBsAg combined with HBV DNA decrease of less than 2 log10 IU/mL are noted at week 12 of treatment [107]. Thus, a stopping rule or motivation for continuing peg-IFN treatment has been established for HBeAg negative patients, based on qHBsAg and HBV DNA measurements at baseline and 12 weeks after the initiation of treatment [108]. Additionally, a second stopping rule at 24 weeks in cases of HBsAg > 7500 IU/mL has been proposed to increase the cost effectiveness of treatment in HBeAg-negative patients with E genotype [109]. In a Greek study with patients infected with genotype D virus, a qHBsAg decline of greater than 10% at week 24 of peg-IFN treatment was associated with SR and when combined with the 12 week stopping rule almost two-thirds of patients who did not achieve SR could be identified [110]. 

In combination treatment with Peg-IFN and adefovir, HBeAg(-) patients who responded to therapy were shown to have lower HBV-RNA levels than non-responders at all time points, there was an independent association of low pretreatment HBV RNA and response to Peg-IFN and adefovir (OD: 0.44; *p* = 0.019) and an earlier and steeper HBV RNA decline in the group of responders (*p* = 0.028) [62]. 

#### 3.4.2. Predictive Biomarkers for Functional Cure with Nucleos(t)ide Analog Treatment

The third generation NAs are potent and with high genetic barrier, therefore able in the majority of cases to lead to viral suppression with undetectable HBV DNA in serum. In this case, the prediction of NA treatment-related HBsAg clearance (the most closely related to cure outcome) has become an important issue, although it is rare and accomplished after long duration of therapy [111,112]. 

Absolute values of qHBsAg could be an important predictive marker in HBeAg(-) NA treated infection, as low levels at baseline (<730–1000 IU/mL) have been associated with functional cure [113,114]. Also, the kinetics of qHBsAg during NA treatment has been studied. An early decline in qHBsAg at six months of therapy [115], on-treatment yearly reductions > 0.166 log IU/mL [105], and a >0.5 log IU/mL drop in the two years after viral response [116] were found to be predictive of subsequent HBsAg clearance.

In a recent study, a qHBsAg reduction > 0.3 log IU/mL in three years of NA treatment had positive and negative predictive values of 42% and 100% respectively, for the identification of patients achieving low levels of HBsAg (<120 IU/mL). The annual decline of qHBsAg was also greater in patients achieving low HBsAg levels (−0.257) than in those who did not (−0.057), (*p* < 0.001). No baseline variables predicted functional cure under NA treatment [117].

#### 3.4.3. Predictive Biomarkers for Discontinuation of Nucleos(t)ide Analogs 

As stated in the recent EASL guidelines, NAs in HBeAg(-) patients should be administered long term until HBsAg loss, but in selected cases of non-cirrhotic patients stopping treatment could be considered after long term (>3 years) of on treatment complete viral response, but only if close post-NA monitoring is feasible [40]. According to previous guidelines of the Asian Pacific Association for the Study of the Liver (APASL), cessation of NAs can be considered in HBeAg(-) hepatitis, if HBV DNA has been undetectable on three separate measurements, each at least six months apart [118,119]. 

When NA treatment is discontinued, sustained off-therapy virological response is defined based on serum HBV DNA levels, which according to EASL 2017 guidelines should be less than 2000 IU/mL for at least 12 months after end of treatment (EOT). In the majority of the cases, after stopping NAs, HBV DNA becomes detectable in serum accompanied or not with increased aminotransferases. This flare can be benign, even beneficial and in fact, following cessation of NA therapy, a high percentage of patients achieve HBsAg clearance after a transient increase in ALT and/or detectable viremia [120,121,122]. This is probably due to the restoration of the immune system which becomes capable of clearing the replicating virus after the inhibition of the viral reverse transcriptase is withdrawn [123,124]. 

HBsAg levels at EOT appear to be an important predictor of HBsAg loss. In HBeAg negative patients qHBsAg < 100 IU/mL at EOT has been found to be a significant independent factor for subsequent functional cure [120,122,125]. Moreover, it was recently suggested that close qHBsAg monitoring, at least every three months and more frequently according to ALT, after stopping NA therapy, is important for the differentiation of benign flare that could lead to HBsAg seroclearance, from a flare that would lead to further liver deterioration and needs to be treated. Decreasing qHBsAg after the ALT peak appears to be related with benign flares and in those patients re-treatment could be withheld [126].

On the other hand, prediction of virological and clinical relapse after NA discontinuation is also extremely valuable. In an Asian study [56], including HBeAg(-) patients, serum HBV RNA levels were found to be significantly associated with virological relapse after NA discontinuation. In one study that included 33 patients who discontinued NA treatment after a long period of undetectable serum HBV DNA, it was found that at the EOT 21 patients (63.64%) were serum HBV RNA positive. Viral relapse was seen in 21 (100%) of the HBV RNA positive versus 3 (25%) of the 12 patients with undetectable HBV RNA at the EOT (*p* = 0.001) [63]. Although these results are promising, it needs to be noted that the majority of HBeAg(-) patients under effective NA treatment become serum HBV RNA negative [62]. Nevertheless, it appears that serum HBV RNA may prove to be a predictive biomarker for the safe discontinuation of NA therapy, primarily as an indicator of virological and possibly clinical relapse, since the detection HBV RNA in serum is associated with the risk of viral rebound.

### 3.5. Hepatocellular Carcinoma

Hepatocellular carcinoma (HCC) is a frequent and life-threatening complication of HBV chronic infection even in HBe antigen negative phases [127]. Liver cirrhosis and HBV viremia (positive HBV DNA) are the critical factors for the development of HCC in chronically infected individuals, even on long-term antiviral treatment [128]. In fact, low level viremia (<2000 IU/mL) under treatment with potent NAs has been associated with a higher risk of HCC than in maintained complete virological response (HR 1.98, 95% CI 1.28–3.06, *p* = 0.002) [129].

Although in cases of HCC, serum HBV DNA levels do not have a strong association with intrahepatic viral load, which also differs between the areas of tumor and surrounding non tumor tissue [130], the level of HBV viremia has been shown to be an independent HCC risk factor (HBV DNA ≥ 5.0 vs. < 5 log IU/mL, HR 3.78, 95% CI 1.20–11.9, *p* < 0.02) [131].

In a relatively recent meta-analysis, high qHBsAg was found to be associated with HCC. Unfortunately, pooled data from only two studies that fulfilled the criteria were used and showed that the risk of HCC occurrence in patients with high HBsAg levels compared to low HBsAg levels [132,133] was significant (OR: 2.21; 95% CI, 1.52–3.22; *p* < 0.01) [134].

More recently, in a group of CHB patients who were not treated with NAs several viral biomarkers including HBV DNA, HBV genotype and HBcrAg, but not qHBsAg, were found to be associated with the incidence of HCC. Specifically, HBcrAg was independently associated with HCC development at levels > 2.9 log U/mL (HR, 5.05; 95% confidence interval (CI), 2.40–10.63) was better predictive marker than HBV DNA [135]. In another study involving patients with undetectable HBV DNA due to effective antiviral therapy, HBcrAg ≥ 7.8 kU/mL was also found to be predictive for HCC with OR 3.27 in the total group and higher at 5.95 in non cirrhotics [136]. 

### 3.6. Future Therapies and Viral Biomarkers

Current treatment of HBeAg negative chronic hepatitis with potent NAs with high genetic barrier for resistance, is highly effective in terms of viral suppression but does not lead to viral elimination. New drugs and therapeutic strategies are emerging that target different steps of the virus life cycle and enhance the immune response against HBV, aiming at complete viral clearance [137]. It is obvious that treatment monitoring and evaluation of response to future therapies would require increased sensitivity of the current assays of viral molecular markers, for a negative result to reflect complete viral eradication from the liver. Moreover, according to the mode of action of the drug (inhibition of viral entry, capsid formation, gene expression, cccDNA formation and stability, etc.) [138] it is possible that other drug specific viral biomarkers will emerge.

## 4. Conclusions

Viral specific biomarkers are used for the diagnosis and monitoring of HBeAg negative hepatitis. Serology and HBV DNA quantification are the established and widely used tests. The implementation of HBsAg quantification is expanding for monitoring the disease in its natural course and on treatment. Other biomarkers, including HBcrAg and HBV RNA, are still under evaluation.

## Figures and Tables

**Table 1:** Hepatitis B Virus (HBV) serology interpretation.

HBsAg	Total Anti-HBc	IgM Anti-HBc	Anti-HBs	Interpretation
-	-	-	-	No HBV infection—susceptible
-	-	-	+ ^1^	HBV immune—vaccinated
-	+	-	+	Past HBV infection
+	+	+	-	Acute HBV infection
+	+	-	-	Chronic HBV infection
-	+	-	-	Inconclusive ^2^

^1^ anti-HBs > 10 mIU/mL; ^2^ Past Infection (more common), False positive anti-HBc, Occult HBV infection. HBsAg: hepatitis B surface antigen; Anti-HBc: hepatitis B core antibody; anti-HBs: hepatitis B surface antibody.

**Table 2:** HBV virological on treatment monitoring and evaluation of results.

	HBV DNA	Comment
Primary non-response	Drop < 1 log10 IU/mL	Treatment week 12
Partial virological response	Detectable and drop > 1 log10 IU/mL)	Treatment week 24 (Low GB NA) or 48 (High GB NA)
Complete virological response	Undetectable	Treatment week 48
Viral breakthrough ^1^	Increase >1 log_10_ IU/mL compared to nadir achieved during treatment, or detectable > 100 IU/mL when previously undetectable	Confirmed in 2 measurements 1 month apart
Functional cure ^2^	Undetectable and HBsAg negative	Anti-HBs +/-

GB: genetic barrier; NA: nucleos(t)ide analog; ^1^ In virological breakthrough, treatment compliance should be considered. In compliant patients virological breakthrough is related to HBV drug-resistance; ^2^ HBsAg seroconversion is considered the optimal endpoint of treatment, even if it does not correspond to complete viral clearance and covalently closed circular DNA (cccDNA), while in low levels, is still found present in infected hepatocytes.
